# Using Machine Learning Approaches on Dynamic Patient-Reported Outcomes to Cluster Cancer Treatment-Related Symptoms

**DOI:** 10.3390/curroncol32060334

**Published:** 2025-06-06

**Authors:** Nora Asper, Hans Friedrich Witschel, Louise von Stockar, Emanuele Laurenzi, Hans Christian Kolberg, Marcus Vetter, Sven Roth, Gerd Kullak-Ublick, Andreas Trojan

**Affiliations:** 1 Center for Dental Medicine and Faculty of Medicine, University of Zurich, 8032 Zurich, Switzerland; hawtrey@outlook.de; 2 School of Business, FHNW, University of Applied Sciences and Arts Northwestern Switzerland, 4600 Olten, Switzerland; hansfriedrich.witschel@fhnw.ch (H.F.W.); emanuele.laurenzi@fhnw.ch (E.L.); 3 Mobile Health AG, 8008 Zurich, Switzerland; louise.vonstockar@mobilehealth.ch; 4 Marienhospital Bottrop GmbH, 46236 Bottrop, Germany; hans-christian.kolberg@mhb-bottrop.de; 5 Cantonal Hospital Baselland, 4410 Liestal, Switzerland; marcus.vetter@ksbl.ch; 6 Department of Clinical Pharmacology and Toxicology, University Hospital Zurich, University of Zurich, 8006 Zurich, Switzerland; gerd.kullak@usz.ch; 7 BreastCenter Zürichsee, 8810 Horgen, Switzerland

**Keywords:** electronic patient-reported outcome (ePRO), symptom cluster, cancer, eHealth, real world evidence, adherence, decision support, machine learning

## Abstract

In patients undergoing systemic treatment for cancer, symptom tracking via electronic patient-reported outcomes (ePROs) has been used to optimize communication and monitoring, and facilitate the early detection of adverse effects and to compare the side effects of similar drugs. We aimed to examine whether the patterns in electronic patient-reported outcomes, without any additional clinician data input, are predictive of the underlying cancer type and reflect tumor- and treatment-associated symptom clusters (SCs). The data were derived from a total of 226 patients who self-reported on the presence and severity (according to the Common Terminology Criteria for Adverse Events (CTCAEs)) of more than 90 available symptoms via the medidux^TM^ app (versions 2.0 and 3.2, developed by mobile Health AG based in Zurich, Switzerland). Among these, 172 had breast cancer as the primary tumor, 19 had lung, 16 had gut, 12 had blood–lymph, and 7 had prostate cancer. For this secondary analysis, a subgroup of 25 patients with breast cancer were randomly selected to reduce the risk of overfitting. The symptoms were aggregated by counting the days on which a particular symptom was reported, resulting in a symptom vector for each patient. A logistic regression model was trained to predict the type of the respective tumor from the symptom vectors, and the symptoms with coefficients above (0.1) were graphically displayed. The machine learning model was not able to recognize any of the patients with prostate and blood–lymph cancer, likely as these cancer types were barely represented in the dataset. The Area Under the Curve (AUC) values for the three remaining cancer types were breast cancer: 0.74 (95% CI [0.624, 0.848]); gut cancer: 0.78 (95% CI [0.659, 0.893]); and lung cancer: 0.63 (95% CI [0.495, 0.771]). Despite the small datasets, for the breast and gut cancers, the respective models demonstrated a fair predictive performance (AUC > 0.7). The generalization of the findings are limited especially due to the heterogeneity of the dataset. This line of research could be especially interesting to monitor individual treatment trajectories. Deviations in the electronic patient-reported symptoms from the treatment-associated symptom patterns could dynamically indicate treatment non-adherence or lower treatment efficacy, without clinician input or additional costs. Similar analyses on larger patient cohorts are needed to validate these preliminary findings and to identify specific and robust treatment profiles.

## 1. Introduction

Cancer is a societal and economic problem, leading to 9.7 million deaths in 2022 [1] and presenting a severe burden for people’s physical and psychological well-being [2]. Although the cancer diagnostics and treatment options have progressed rapidly in recent years, digital symptom reporting and specific symptom management are progressing slowly [3]. Some studies indicate that specific symptoms tend to present concurrently and can be grouped into symptom clusters (SCs) [4,5]. SCs involve the co-occurrence of at least two symptoms and are distinct from other SCs, meaning that the correlation between symptoms within a single symptom cluster is sturdier than the correlations between symptoms in different symptom clusters [4,6]. SCs are informative as the co-occurrence of symptoms can hint towards shared underlying mechanisms and are, for example, used in psychiatry to classify disorders [6]. In cancer, one of the most frequently described symptom clusters is the gastrointestinal SC, consisting of vomiting, general nausea, and a lack of appetite [5,7,8].

Two typical approaches have been used to cluster symptoms. Clinically determined SCs are based on physicians’ observation of symptom co-occurrence or based on the primary cancer site. Statistically determined SCs are purely based on the cluster analyses of symptom distributions [9]. However, patients’ subjective experiences have rarely been considered in the data [9,10]. Recent studies [10,11,12,13] performed surveys and interviews to create SCs based on how patients subjectively interpret their symptoms, perceive the relationships between them, and assess their impact on their quality of life. The individual variability found in the patient-reported SCs significantly differs from the SCs derived from observational studies, multi-symptom assessment tools, and symptom checklists [10]. Patient-centered analyses support the development of more effective and individualized treatment plans, as objectively similar symptom profiles may require different treatment strategies based on personal interpretation and prioritization [9,10]. To improve access to treatment and create more precise and meaningful symptom clusters, smartphone applications (apps) can be used to capture real-time patient-reported data. Electronic patient-reported outcomes (ePROs) have generally been found to improve their quality of care through optimized communication and monitoring [14,15], and the continuous evaluation of the collected data has the potential to predict adverse events [16,17,18].

Recently, clustering approaches have turned to machine learning (ML) techniques to identify more complex patterns in larger datasets [19,20]. Unlike the traditional methods that depend on predefined symptom relationships, ML algorithms can autonomously learn patterns from the data itself [21]. One way ML has been applied is in reverse clustering. As opposed to classical symptom clustering, reverse clustering seeks to reproduce a known dataset. As such, patients are grouped by their existing diagnosis rather than into symptom profiles, and the coefficients are calculated accordingly. Reverse clustering is a way to validate the existing symptom clusters and assess whether symptom clusters accurately represent the population. Reverse clustering has been used in data science, but not yet been applied in the medical field [22] and could improve our understanding of how symptom clusters manifest in different types of cancer and treatments [8,23,24]. However, despite the advances towards more patient-centered symptom clustering and the increasing use of ePROs, dynamic and real-time ePRO data have rarely been leveraged for symptom clustering purposes. Exploring new approaches that combine dynamic ePRO data with ML methods could improve our understanding and characterization of symptom patterns across different cancer types and treatments.

Given the increasing complexity and chronicity of cancer treatments, along with demographic shifts and limited healthcare resources, there is an increasing drive to support oncology through digital innovation. Despite this, tools for systematically and continuously capturing patients’ well-being, symptoms, and vital parameters during and beyond treatment are not yet widely established. Using the patient-centered app medidux™ as an example, this study demonstrates how the systematic and structured digital documentation of their physical complaints, well-being, cognitive functioning, and vital signs can contribute to improving the quality and efficiency of cancer research and care.

In this study, we sought to examine whether the analysis of electronic patient-reported symptoms could “predict” the diagnosed underlying cancer type and confirm tumor- and treatment-associated specific symptom clusters. For this, we used electronic patient-reported outcomes (ePROs) data previously collected through a Class I medical device application. In previous work, this app demonstrated a high level of agreement between the patients and the physicians in their symptom ratings [25] and has been used to compare the performances of biosimilars [26] and investigate symptom patterns indicative of emergency hospital visits [18].

## 2. Materials and Methods

### 2.1. Study Design

This study involves the secondary analysis of 226 patients undergoing treatment, who self-reported the presence and severity of over 90 symptoms using the medidux^TM^ app (formerly Consilium Care) in accordance with the Common Terminology Criteria for Adverse Events (CTCAEs). The patient data were obtained from two studies that both received approval from the Swiss Institutional Review Board (KEK-ZH: 2021-D0051; KEK-ZH: 2017-02028) and were conducted in accordance with the current principles of the Declaration of Helsinki. Additionally, the study is registered on ClinicalTrials.gov (NCT05234021 and NCT03578731) and on the Swiss National Clinical Trials Portal (SNCTP000004711).

### 2.2. Participants

For the original 226 patients, the primary tumors included 172 breast, 19 lung, 16 gut, 12 blood–lymphoma, and 7 prostate cancer cases. For balanced analysis and to reduce the risk of overfitting, 25 patients treated for breast cancer were randomly selected. The eligible participants had initiated adjuvant or neoadjuvant systemic therapy, including with at least one cytotoxic drug +/− antibody treatment, antibody drug conjugate, or antihormone, were over 18 years old; owned a smartphone, spoke German; and had provided written informed consent. From the 226 individuals in this study, 34 were male, 191 were female, and 1 classified as other. The mean age of the participants was 58.4 years (Table 1).

### 2.3. Mobile App

The medidux^TM^ app (formerly consilium care; versions 2.0 and 3.2, developed by mobile Health AG based in Zurich, Switzerland) used in this study is a patient-centered CE-marked medical device software application designed as a digital companion during cancer therapy. Rather than repeatedly requiring patients to fill in digital questionnaires, the app allows for patients to dynamically record their relevant symptoms according to the CTCAE [25]. Aside from over 90 available symptoms, the app also offers cognitive testing and functionalities to record patients’ well-being, vital parameters, and medications. Graphical representations of the recorded data are available for patients and care teams, as well as alerts in case the symptoms exceed grade 2 according to the CTCAE. During the study period of 84 days, the patients were encouraged to enter their individual symptoms daily as they occurred.

### 2.4. Statistical Analyses

For statistical analyses, the symptoms were displayed as a vector for every patient by counting the days during the study period on which a particular symptom was reported. Logistic regression models were fitted for each cancer type to predict the presence or absence of that specific type of cancer based on the corresponding symptom vector of the patient. While logistic regression models are relatively simple, more sophisticated machine learning (ML) approaches were unwarranted due to the small and heterogenous sample. The focus on human interpretability and simplicity is additionally especially important in the context of clinical translational research to foster trust among clinicians who are unfamiliar with ML approaches [27]. To evaluate performance, each model was 10-fold cross-validated, and the Area Under the Curve (AUC) metric was calculated. The AUC is commonly used to assess performance and validate the predictive accuracy of diagnostic tests. It represents the degree to which a test can differentiate between patients who have a disease and those who do not, with a value of 1 indicating perfect discrimination and 0.5 suggesting a performance that is no better than random guessing [28]. The threshold for acceptable discrimination is typically an AUC value of 0.7 [29]. Further, a simple single-sample hypothesis-testing approach was applied to calculate the corresponding 95% confidence intervals (CIs). The model sensitivity and specificity were calculated.

Additionally, confusion matrices were generated to evaluate how well each model could identify the patients with the respective cancer type. The models were discarded if they failed to correctly identify at least one relevant patient. The large coefficients of each remaining model were extracted, assuming these represent typical symptoms of each cancer type or common adverse effects of the associated therapies. These symptoms were visualized in a symptom cloud to highlight relationships and prevalence.

To address class imbalance, we initially trained the models on the full breast cancer dataset (n = 172) without down sampling. However, this led to a marked prediction bias towards the majority class. Therefore, we conducted the random down sampling of the breast cancer group (n = 25) to achieve a more balanced model. The performance metrics for unbalanced analysis are reported in the Results section.

To contextualize the performance of logistic regression, we additionally evaluated two alternative machine learning models: a decision tree and a gradient boosting model. These models were trained on the same symptom vectors and evaluated using 10-fold cross-validation. The performance was assessed using standard metrics, including the Area Under the Curve (AUC), 95% confidence intervals, sensitivity, and specificity. Detailed performance comparisons are reported in the Results section.

## 3. Results

**Figure 1:**
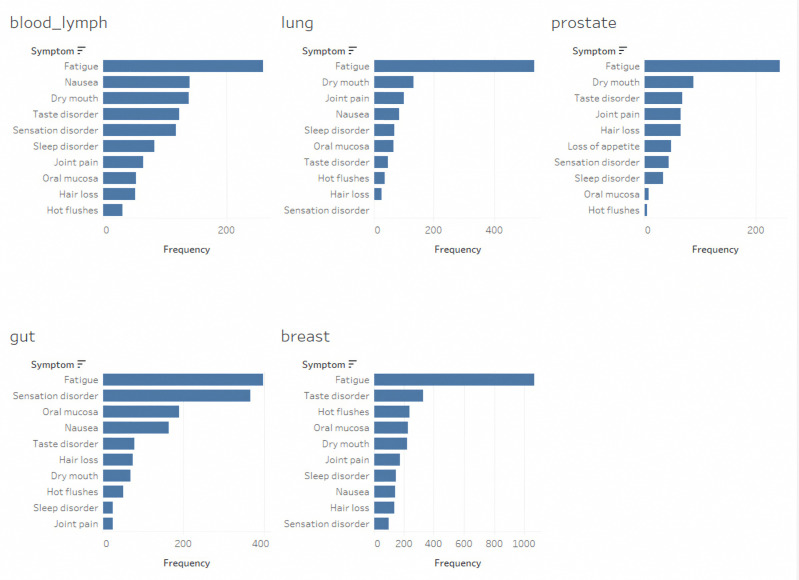
Frequency of 10 most commonly reported symptoms categorized by tumor type.

Over the three-month period, the patients reported between four and sixteen different symptoms [30]. With respect to the ePRO Data, the participants reported a median of 3.0 symptoms daily (with a range of 1.2 to 3.3 symptoms), resulting in a total symptom count of 43,430. The median duration of symptom tracking was 82 days (ranging from 14 to 225 days). The three most frequently reported symptoms varied by cancer type, although fatigue consistently ranked as the most common symptom across all the categories. For the patients with breast cancer, the most reported symptoms included fatigue, hot flashes, and taste disorders. For blood and lymph cancers, fatigue, nausea, and dry mouth were the predominant symptoms. The patients with gut cancer reported fatigue, sensory disorders, and issues with the oral mucosa. The patients diagnosed with prostate cancer experienced fatigue, a dry mouth, and taste disorders (Figure 1).

Although the symptom clusters could potentially be broken down by specific treatment regimens, this analysis was not available from the current dataset due to limited the number of events and the heterogeneity of treatment regimens for the most frequently applied treatments for cancers of the breast, lung, and gut (listed in Table 2).

**Figure 2:**
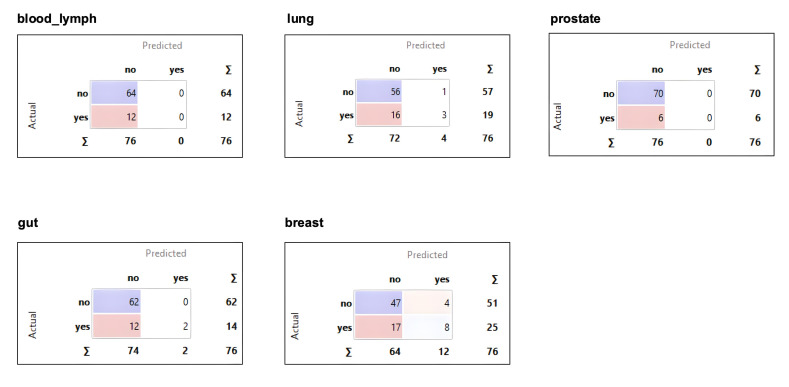
Confusions matrices for all five tumor types.

As illustrated from the confusion matrices (Figure 2), the machine learning model was not able to recognize any patients with prostate or blood–lymph cancers. Consequently, these cancer types were excluded from further analysis. The AUC values and the corresponding 95% confidence intervals for the three remaining cancer types were breast cancer: 0.74 (95% CI [0.62, 0.85]); gut cancer: 0.78 (95% CI [0.66, 0.89]); and lung cancer: 0.63 (95% CI [0.50, 0.77]). All the models show low sensitivity (32%, 14%, and 16%, respectively), but high specificity (92%, 100%, and 98%, respectively). The AUC for lung cancer was not statistically significant as the 95% confidence interval includes 0.5; for the other two cancer types, the confidence intervals indicate a fair performance.

In the preliminary analysis, we trained a logistic regression model on the full set of 172 patients with breast cancer without down sampling. While the model achieved a high sensitivity of 94% for breast cancer, for the other cancer types, sensitivity dropped to 14% for gut cancer and 5% for lung cancer. The AUC values also reflected this imbalance, with 0.65 (95% CI [0.54, 0.76]) for breast, 0.78 (95% CI [0.68, 0.88]) for gut, and 0.65 (95% CI [0.53, 0.77]) for lung cancer. These results demonstrated a strong prediction bias toward breast cancer and justify the application of down sampling in the final analysis to improve the sensitivity of the models.

To evaluate the model performance across the different classifiers, we assessed a decision tree and a gradient boosting model alongside logistic regression. For breast cancer, the AUCs and the corresponding 95% confidence intervals were 0.68 (95% CI [0.56, 0.80]) for the decision tree and 0.69 (95% CI [0.57, 0.81]) for gradient boosting. For gut cancer, the AUCs were 0.51 (95% CI [0.34, 0.68]) and 0.54 (95% CI [0.37, 0.71]), respectively. For lung cancer, both alternatives outperformed logistic regression, achieving 0.72 (95% CI [0.60, 0.84]) with the decision tree and 0.76 (95% CI [0.65, 0.87]) with gradient boosting.

While gradient boosting achieved the best overall performance for lung cancer, logistic regression showed a superior or comparable performance in breast and gut cancers, while also offering better human interpretability. Given this trade-off, logistic regression was selected as the primary model.

**Figure 3:**
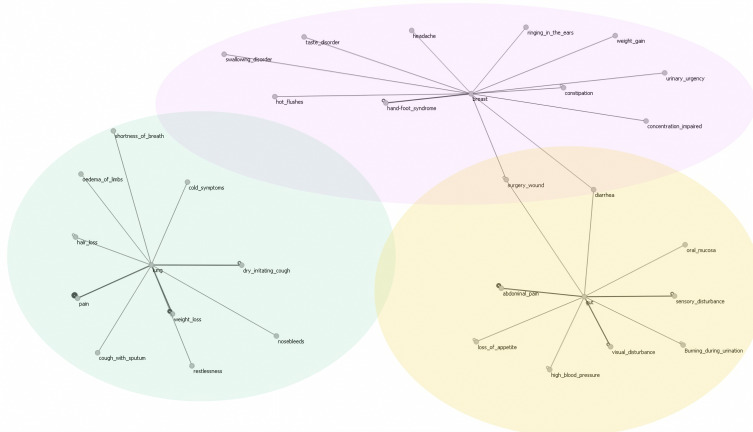
Comprehensive cloud aggregation of symptoms for breast, lung, and gut cancers.

For the three cancer types, large coefficients are graphically displayed in a symptom cloud to illustrate their associations. In this graphical representation, the thicker lines signify higher coefficients, indicating a stronger association (Figure 3).

## 4. Discussion

Our study aimed to explore the potential of the reverse clustering of treatment-related ePROs to predict the underlying cancer types and to confirm the tumor- and treatment-associated symptom clusters. Despite the small participant number, we demonstrated fair AUCs for breast and gut cancers. The models for prostate and blood–lymph cancers performed the worst, not correctly identifying a single patient with the relevant cancer type; however, for these two cancer types, the fewest patients were included (7 and 12, respectively), which likely caused the poor model performance. The breast and gut cancer models nevertheless exhibited an acceptable performance with the data derived from only 25 and 16 patients, demonstrating that exploratory ML studies are feasible even with comparable small ePRO datasets.

Despite not reaching statistical significance, the symptom cloud based on the lung cancer model appears plausible from a medical perspective, suggesting that further investigation may be warranted, as symptom clusters show strong similarity with those from other studies, with all the symptoms except nosebleeds also represented in these SCs [31,32]. Considering that all the symptoms were individually selected, rather than pre-specified through a questionnaire, the data analyzed are inherently informative. Likewise, our models for the SCs in breast and gut cancers overlap with the SCs throughout the literature, particularly for symptom clusters such as fatigue and pain for both the cancer types and gastrointestinal symptoms, especially for gut cancer [23]. In addition, for the patients with gut cancer, our model additionally identified clusters involving sensory disturbances, high blood pressure, visual disturbances, and burning during urination. These symptoms are less frequently emphasized in prior symptom cluster studies [33], potentially reflecting the influence of comorbidities such as diabetes mellitus, which is often prevalent in this patient population, but not always explicitly accounted for in previous studies. In breast cancer, fatigue, sleep disturbances, and taste disorders clustered also as expected, which is consistent with prior reports [23,34]. Interestingly, our breast cancer model also revealed symptoms such as headaches and ringing in the ears in the symptom cloud. As these symptoms are not typically highlighted in the previous studies [23,34], their emergence may reflect therapy-associated neurotoxicity or previously under-recognized symptom constellations, thus potentially offering new hypotheses for further clinical investigation. Although SCs are likely to be influenced by specific treatment regimens (Table 2), this analysis was not conducted on the current dataset. Thus, it is important to note that from a human-interpretable perspective, it seems likely that the identified symptom clusters might reflect treatment-related side effects rather than malignancy-specific symptoms alone. This interpretation is further supported by our comparison with prior studies, where many of the identified clusters correspond to known therapy-associated symptom patterns.

So far, ML approaches have already been applied in various areas of medicine, including radiology for the detection of tumors [35,36] and in cardiology, where ML models assist in cardiac image detection and the classification or prediction of hypertension [37,38]. The novelty of our approach lies in the fact that no clinical data was required since the dynamic symptom datasets were exclusively reported by empowered patients. Given larger samples sizes, it seems likely that robust treatment-specific symptom clusters can be derived from such ePRO data; this would allow for the improved monitoring of treatment trajectories, with deviations from those symptom patterns possibly acting as indicators for treatment non-adherence or lower treatment efficacy. In terms of adherence, if certain symptoms are no longer reported, this might indicate that medication is either routinely forgotten or that the patient has intentionally stopped their intake to mitigate a high side effect burden [39]. Alternatively, persistent symptom changes could also reflect reduced treatment efficacy, warranting further clinical assessment. If indicated by changes in an individual symptom cluster, physicians could have a conversation with their patients to identify the cause of non-adherence and either adjust their treatment or support them in building treatment habits. The earlier detection of such deviations could help timely and proactive clinical interventions. Integrating symptom-based prediction models and results into digital monitoring tools and clinical practice could help prioritize the patients at a high risk of non-adherence, eventually improving treatment success and potentially reducing hospitalizations [15,18]. Such clinical applications underline the potential value of the proposed prediction model beyond the statistical performance. Studies based on ePRO data may also improve the representation of elderly patients by increasing accessibility, improving patient engagement, and facilitating data collection for decentralized clinical trials (DCTs).

There are several limiting factors to this study. Reverse clustering has challenges such as the need for reliable and consistent initial data, as deviations in the initial entries could impede the formation of valid symptom clusters. The small sample size per cancer type, heterogeneity, and lack of detailed information on differential therapies, as well as on comorbidities, limits the generalizability of our findings and may lead to overfitting. To mitigate class imbalance, the breast cancer cohort was down sampled. While this limited the ability to fully capture symptom heterogeneity within the breast cancer group, it improved the model sensitivity to other cancer types. Due to the nature of the secondary dataset and comparable small sample size, detailed subgroup information regarding chemotherapy, antibody treatments, and radiotherapy could not be retrieved. All the models were strongly conservative and generally tended to reject the patients, which led to a large proportion of false negative cases. The high threshold likely led to fewer symptoms being identified within each symptom cluster, but resulted in clusters that were more clearly distinguishable between the different tumor entities. While low sensitivity indicates that the models at this performance level would not be suitable for screening purposes, they could have value for individual patients undergoing treatment. The typical symptom clusters could be used to detect changes at an individual level and helps distinguish them from random fluctuations.

The fair AUC value for the breast and gut cancer models indicates that relevant patterns could be extracted from the data, although larger datasets will be needed to train and validate more robust models and potentially explore the combination of ePROs with clinical markers in a hybrid approach. External validation on independent datasets was not performed, as no independent dataset was available, which limits the generalizability of our findings. Future work could explore advanced deep neural networks for clustering, including generative models based on Riemannian geometry and fuzzy clustering combined with graph convolutional networks [40,41]. Since we performed the secondary analysis of an existing dataset, a formal sample size calculation or post hoc power analysis was not conducted. However, to our knowledge, this is the first study to introduce the concept of reverse clustering into the medical field.

## 5. Conclusions

In conclusion, this exploratory study explores the potential of symptom clusters based on treatment-related ePRO data to retrospectively “identify” the type of underlying cancer using treatment-related symptoms. Despite the small dataset, our breast and gut cancer models demonstrated a fair performance and overlap with the symptom clusters derived from patient-reported outcomes in the existing literature, but further clinical investigation is required.

## Figures and Tables

**Table 1:** Baseline characteristics.

	Count	Percentage (%)
**Overall**	226	100
**Primary Tumor**		
Breast Cancer	172	76.1
Lung Cancer	19	8.4
Gut Cancer	16	7.1
Blood–lymph Cancer	12	5.3
Prostate Cancer	7	3.1
**Gender**		
Male	34	15
Female	191	84.5
Diverse	1	0.4
**Mean Age**	58.4	
**Selected Patients for Analysis**	60	
**Primary Tumor**		
Breast Cancer	25	41.7
Lung Cancer	19	31.7
Gut Cancer	16	26.7
**Gender**		
Male	15	25
Female	45	75
Diverse	0	0
**Mean Age**	50	

**Table 2:** Most frequently applied treatments for cancers of breast, lung, and gut in percentage.

Most Frequently Applied Therapies	Percentage (%)
Herceptin/Perjeta +/− Docetaxel/Carboplatin	16.3
Antihormone +/− Everolimus o. CDK4/6-Inhibitor	16.3
Paclitaxel +/− Carboplatin	12.1
Docetaxel-Endoxan +/− Antihormon	10.6
EC-Paclitaxel	9.9
Checkpointinhibitor +/− Chemo	8.5
Capecitabine	6.4
EC-Docetaxel	6.4
Docetaxel/Carboplatin	2.8
Platine + Pemetrexed	2.1
FOLFIRI	2.1
CAPOX	2.1
Docetaxel	1.4
Platine + Etoposid	1.4
FOLFOX	1.4

## Data Availability

Data were available from the medical device patient app. All data generated or analyzed during this study are included in this article. Further enquiries can be directed to the corresponding author on reasonable request.

## Abbreviations

The following abbreviations are used in this manuscript:

APP	Application
AUC	Area Under the Curve
CI	Confidence Interval
CTCAE	Common Terminology Criteria for Adverse Event
DCTs	Decentralized Clinical Trials
ePROs	Electronic Patient-Reported Outcomes
ML	Machine Learning
SCs	Symptom Clusters

## References

[1] Bray F., Laversanne M., Sung H., Ferlay J., Siegel R.L., Soerjomataram I., Jemal A. (2024). Global Cancer Statistics 2022: GLOBOCAN Estimates of Incidence and Mortality Worldwide for 36 Cancers in 185 Countries. CA A Cancer J. Clin..

[2] Lee E.M., Jiménez-Fonseca P., Galán-Moral R., Coca-Membribes S., Fernández-Montes A., Sorribes E., García-Torralba E., Puntí-Brun L., Gil-Raga M., Cano-Cano J. (2023). Toxicities and Quality of Life during Cancer Treatment in Advanced Solid Tumors. Curr. Oncol..

[3] de Góes Salvetti M., Sanches M.B. (2022). Symptom Cluster: Management and Advanced Practices in Oncology Nursing. Rev. Esc. Enferm. USP.

[4] Kirkova J., Walsh D., Aktas A., Davis M.P. (2010). Cancer Symptom Clusters: Old Concept but New Data. Am. J. Hosp. Palliat. Care.

[5] Kwekkeboom K.L. (2016). Cancer Symptom Cluster Management. Semin. Oncol. Nurs..

[6] Kim H.-J., McGuire D.B., Tulman L., Barsevick A.M. (2005). Symptom Clusters: Concept Analysis and Clinical Implications for Cancer Nursing. Cancer Nurs..

[7] Cheung W.Y., Le L.W., Zimmermann C. (2009). Symptom Clusters in Patients with Advanced Cancers. Support. Care Cancer.

[8] Dong S.T., Butow P.N., Costa D.S.J., Lovell M.R., Agar M. (2014). Symptom Clusters in Patients with Advanced Cancer: A Systematic Review of Observational Studies. J. Pain Symptom Manag..

[9] Kirkova J., Aktas A., Walsh D., Davis M.P. (2011). Cancer Symptom Clusters: Clinical and Research Methodology. J. Palliat. Med..

[10] Kwekkeboom K.L., Wieben A., Braithwaite L., Hopfensperger K., Kim K.S., Montgomery K., Reske M., Stevens J. (2022). Characteristics of Cancer Symptom Clusters Reported through a Patient-Centered Symptom Cluster Assessment. West. J. Nurs. Res..

[11] Dong S.T., Butow P.N., Tong A., Agar M., Boyle F., Forster B.C., Stockler M., Lovell M.R. (2016). Patients’ Experiences and Perspectives of Multiple Concurrent Symptoms in Advanced Cancer: A Semi-Structured Interview Study. Support. Care Cancer.

[12] Erickson J.M., Ameringer S., Linder L., Macpherson C.F., Elswick R.K., Luebke J.M., Stegenga K. (2019). Using a Heuristic App to Improve Symptom Self-Management in Adolescents and Young Adults with Cancer. J. Adolesc. Young Adult Oncol..

[13] Macpherson C.F., Linder L.A., Ameringer S., Erickson J., Stegenga K., Woods N.F. (2014). Feasibility and Acceptability of an iPad Application to Explore Symptom Clusters in Adolescents and Young Adults with Cancer. Pediatr. Blood Cancer.

[14] Salmani H., Nasiri S., Ahmadi M. (2024). The Advantages, Disadvantages, Threats, and Opportunities of Electronic Patient-Reported Outcome Systems in Cancer: A Systematic Review. Digit. Health.

[15] Trojan A., Kühne C., Kiessling M., Schumacher J., Dröse S., Singer C., Jackisch C., Thomssen C., Kullak-Ublick G.A. (2024). Impact of Electronic Patient-Reported Outcomes on Unplanned Consultations and Hospitalizations in Patients with Cancer Undergoing Systemic Therapy: Results of a Patient-Reported Outcome Study Compared with Matched Retrospective Data. JMIR Form. Res..

[16] Avery K.N.L., Richards H.S., Portal A., Reed T., Harding R., Carter R., Bamforth L., Absolom K., O’Connell Francischetto E., Velikova G. (2019). Developing a Real-Time Electronic Symptom Monitoring System for Patients after Discharge Following Cancer-Related Surgery. BMC Cancer.

[17] Holch P., Warrington L., Bamforth L.C.A., Keding A., Ziegler L.E., Absolom K., Hector C., Harley C., Johnson O., Hall G. (2017). Development of an Integrated Electronic Platform for Patient Self-Report and Management of Adverse Events during Cancer Treatment. Ann. Oncol..

[18] Trojan A., Laurenzi E., Roth S., Kiessling M., Atassi Z., Kadvany Y., Mannhart M., Witschel H.F., Jüngling S., Kullak-Ublick G.A. (2024). Towards an Early Warning System for Monitoring of Cancer Patients Using Hybrid Interactive Machine Learning. Front. Digit. Health.

[19] LeCun Y., Bengio Y., Hinton G. (2015). Deep Learning. Nature.

[20] Tseng H.-H., Wei L., Cui S., Luo Y., Ten Haken R.K., El Naqa I. (2018). Machine Learning and Imaging Informatics in Oncology. Oncology.

[21] Mitchell T. (1997). Machine Learning.

[22] Owsiński J.W., Opara K., Stańczak J., Kacprzyk J., Zadrożny S. (2017). Reverse Clustering: An Outline for a Concept and Its Use. Toxicol. Environ. Chem..

[23] de Rooij B.H., Oerlemans S., van Deun K., Mols F., de Ligt K.M., Husson O., Ezendam N.P.M., Hoedjes M., van de Poll-Franse L.V., Schoormans D. (2021). Symptom Clusters in 1330 Survivors of 7 Cancer Types from the PROFILES Registry: A Network Analysis. Cancer.

[24] Lee L.J., Han C.J., Saligan L., Wallen G.R. (2024). Comparing Symptom Clusters in Cancer Survivors by Cancer Diagnosis: A Latent Class Profile Analysis. Support. Care Cancer.

[25] Trojan A., Leuthold N., Thomssen C., Rody A., Winder T., Jakob A., Egger C., Held U., Jackisch C. (2021). The Effect of Collaborative Reviews of Electronic Patient-Reported Outcomes on the Congruence of Patient-and Clinician-Reported Toxicity in Cancer Patients Receiving Systemic Therapy: Prospective, Multicenter, Observational Clinical Trial. J. Med. Internet Res..

[26] Trojan A., Roth S., Atassi Z., Kiessling M., Zenhaeusern R., Kadvany Y., Schumacher J., Kullak-Ublick G.A., Aapro M., Eniu A. (2024). Comparison of the Real-World Reporting of Symptoms and Well-Being for the HER2-Directed Trastuzumab Biosimilar Ogivri With Registry Data for Herceptin in the Treatment of Breast Cancer: Prospective Observational Study (OGIPRO) of Electronic Patient-Reported Outcomes. JMIR Cancer.

[27] Rudin C. (2019). Stop Explaining Black Box Machine Learning Models for High Stakes Decisions and Use Interpretable Models Instead. Nat. Mach. Intell..

[28] Kleppe A. (2022). Area under the Curve May Hide Poor Generalisation to External Datasets. ESMO Open.

[29] Çorbacıoğlu Ş.K., Aksel G. (2023). Receiver Operating Characteristic Curve Analysis in Diagnostic Accuracy Studies: A Guide to Interpreting the Area under the Curve Value. Turk. J. Emerg. Med..

[30] Trojan A., Huber U., Brauchbar M., Petrausch U. (2020). Consilium Smartphone App for Real-World Electronically Captured Patient-Reported Outcome Monitoring in Cancer Patients Undergoing Anti-PD-L1-Directed Treatment. Case Rep. Oncol..

[31] Li N., Wu J., Zhou J., Wu C., Dong L., Fan W., Zhang J. (2021). Symptom Clusters Change Over Time in Patients With Lung Cancer During Perichemotherapy. Cancer Nurs..

[32] Yang X., Bai J., Liu R., Wang X., Zhang G., Zhu X. (2024). Symptom Clusters and Symptom Network Analysis during Immunotherapy in Lung Cancer Patients. Support. Care Cancer.

[33] Hao J., Gu L., Liu P., Zhang L., Xu H., Qiu Q., Zhang W. (2021). Symptom Clusters in Patients with Colorectal Cancer after Colostomy: A Longitudinal Study in Shanghai. J. Int. Med. Res..

[34] So W.K.W., Law B.M.H., Ng M.S.N., He X., Chan D.N.S., Chan C.W.H., McCarthy A.L. (2021). Symptom Clusters Experienced by Breast Cancer Patients at Various Treatment Stages: A Systematic Review. Cancer Med..

[35] Hosny A., Parmar C., Quackenbush J., Schwartz L.H., Aerts H.J.W.L. (2018). Artificial Intelligence in Radiology. Nat. Rev. Cancer.

[36] Bi W.L., Hosny A., Schabath M.B., Giger M.L., Birkbak N.J., Mehrtash A., Allison T., Arnaout O., Abbosh C., Dunn I.F. (2019). Artificial Intelligence in Cancer Imaging: Clinical Challenges and Applications. CA A Cancer J. Clin..

[37] Silva G.F.S., Fagundes T.P., Teixeira B.C., Chiavegatto Filho A.D.P. (2022). Machine Learning for Hypertension Prediction: A Systematic Review. Curr. Hypertens. Rep..

[38] Jiang B., Guo N., Ge Y., Zhang L., Oudkerk M., Xie X. (2020). Development and Application of Artificial Intelligence in Cardiac Imaging. Br. J. Radiol..

[39] Molloy G.J., Messerli-Bürgy N., Hutton G., Wikman A., Perkins-Porras L., Steptoe A. (2014). Intentional and Unintentional Non-Adherence to Medications Following an Acute Coronary Syndrome: A Longitudinal Study. J. Psychosom. Res..

[40] Sun L., Hu J., Zhou S., Huang Z., Ye J., Peng H., Yu Z., Yu P. (2024). RicciNet: Deep Clustering via A Riemannian Generative Model. Proceedings of the ACM Web Conference 2024.

[41] Yang Y., Li G., Li D., Zhang J., Hu P., Hu L. (2025). Integrating Fuzzy Clustering and Graph Convolution Network to Accurately Identify Clusters From Attributed Graph. IEEE Trans. Netw. Sci. Eng..

